# A comparative analysis of Patient-Reported Expanded Disability Status Scale tools

**DOI:** 10.1177/1352458515616205

**Published:** 2015-11-12

**Authors:** Christian DE Collins, Ben Ivry, James D Bowen, Eric M Cheng, Ruth Dobson, Douglas S Goodin, Jeannette Lechner-Scott, Ludwig Kappos, Ian Galea

**Affiliations:** Clinical Neurosciences, Clinical and Experimental Sciences, Faculty of Medicine, University of Southampton, Southampton General Hospital, Southampton, UK; Clinical Neurosciences, Clinical and Experimental Sciences, Faculty of Medicine, University of Southampton, Southampton General Hospital, Southampton, UK; Multiple Sclerosis Center, Swedish Neuroscience Institute, Seattle, WA, USA; Department of Neurology, David Geffen School of Medicine, VA Greater Los Angeles Healthcare System, University of California, Los Angeles (UCLA), Los Angeles, CA, USA; Blizard Institute, Barts and The London School of Medicine and Dentistry, Queen Mary University of London, London, UK; Department of Neurology, University of California, San Francisco (UCSF), San Francisco, CA, USA; Hunter Medical Research Institute, The University of Newcastle, Australia and Department of Neurology, John Hunter Hospital, Newcastle, NSW, Australia; Departments of Medicine, Clinical Research, Biomedicine and Biomedical Engineering, University Hospital Basel, Basel, Switzerland; Clinical Neurosciences, Clinical and Experimental Sciences, Faculty of Medicine, University of Southampton, Southampton General Hospital, Southampton, UK/Wessex Neurosciences Centre, University Hospital Southampton NHS Foundation Trust, Southampton, UK

**Keywords:** Expanded disability status scale, Kurtzke scale, Neurostatus, multiple sclerosis, Patient-reported Expanded Disability Status Scale, patient reported, self administered

## Abstract

**Background:**

Patient-Reported Expanded Disability Status Scale (PREDSS) tools are an attractive alternative to the Expanded Disability Status Scale (EDSS) during long term or geographically challenging studies, or in pressured clinical service environments.

**Objectives:**

Because the studies reporting these tools have used different metrics to compare the PREDSS and EDSS, we undertook an individual patient data level analysis of all available tools.

**Methods:**

Spearman’s rho and the Bland–Altman method were used to assess correlation and agreement respectively.

**Results:**

A systematic search for validated PREDSS tools covering the full EDSS range identified eight such tools. Individual patient data were available for five PREDSS tools. Excellent correlation was observed between EDSS and PREDSS with all tools. A higher level of agreement was observed with increasing levels of disability. In all tools, the 95% limits of agreement were greater than the minimum EDSS difference considered to be clinically significant. However, the intra-class coefficient was greater than that reported for EDSS raters of mixed seniority. The visual functional system was identified as the most significant predictor of the PREDSS–EDSS difference.

**Conclusion:**

This analysis will (1) enable researchers and service providers to make an informed choice of PREDSS tool, depending on their individual requirements, and (2) facilitate improvement of current PREDSS tools.

## Introduction

Kurtzke introduced the Expanded Disability Status Scale (EDSS) in 1983^1^ as a revision of his initial 1955 Disability Status Scale,^2^ to provide a valid and comprehensive assessment of multiple sclerosis (MS)-related disability, for which it still remains the gold-standard tool despite its limitations.^3^

EDSS scores range from 0 to 10 in 0.5 step intervals, with 0 being no impairment, and 10 being death from MS. At low levels of disability (scores from 0 to 3.5), the EDSS score is determined by neurological examination, while at high levels of disability (scores ⩾ 5.5), it is primarily influenced by ambulation and dependence on help in daily activities. EDSS scores between 4.0 and 5.0 are reached by combinations of neurological examination, functional status and ambulation assessment.

Physician-determined EDSS (henceforth referred to as EDSS) is time-consuming, expensive and restricts assessment of the EDSS to clinic visits, which may be infrequent or impractical. There have been several tools developed to enable patients to report their own EDSS score, that is, a Patient-Reported EDSS (henceforth referred to as PREDSS).^4–11^ PREDSS is potentially useful in various situations such as patient follow-up during long term or geographically challenging studies where clinic attendance is difficult, or to enable EDSS assessment in busy or under-staffed clinical service environments.

There are two clinical scenarios where PREDSS may be employed instead of the EDSS:

1. In the first scenario, PREDSS and EDSS are used interchangeably and therefore it is important to have agreement between the two. In this case, agreement statistics would be relevant. There are several measures of agreement. Percentage agreement is a useful directly intuitive measure, but it does not correct for chance. Cohen’s kappa statistic is the proportion of agreement after having allowed for that expected by chance. The weighted kappa coefficient additionally puts a weight to the distance between disagreements. The value of kappa is dependent on prevalence of the scores within a particular population.^12^ The intra-class coefficient (ICC) measures the proportion of total variance that is due to differences between patients (with the rest being the variance due to differences in the scales being compared); therefore, its size depends on the variability in the sample.^13^ The Bland–Altman method visualizes the data and more openly describes agreement, instead of attempting to summarize agreement as a statistic.^14^ It is now recognized that the Bland–Altman method is the most appropriate way to assess agreement, and as a result, it has become the most frequently used method.^15^ The differences between the two scores are plotted against the reference or ‘gold standard’ method (in this case, the EDSS). Horizontal lines are drawn at the mean difference, and at the 95% limits of agreement, which are defined as the mean difference plus and minus 1.96 times the standard deviation of the mean difference. If the difference between the 95% limits of agreement is not clinically significant, a correction factor (the mean difference) may be used to enable interchangeability between PREDSS and EDSS if the PREDSS consistently underscores or overscores the EDSS. It is accepted that EDSS change is clinically significant if its magnitude is of at least 1.0 point on Kurtzke’s EDSS in patients with an EDSS score of 5.5 or lower, or 0.5 point in patients with a higher EDSS score.^16^
2. In the second scenario, PREDSS is the only tool used to serially assess patients in a clinic or study where it is not so necessary to have agreement of scores between PREDSS and EDSS, but it is important to have a linear relationship between PREDSS and EDSS which is as good as possible with respect to strength and direction. In this case, correlation statistics would be relevant.

It is difficult to compare the PREDSS tools with each other since the original study reports used different metrics to compare PREDSS and EDSS scores. This study aims to make a head-to-head comparison of the tools for which the original individual patient level data was available, thus enabling researchers or clinicians to make a well-informed decision in choosing a PREDSS tool that best suits their needs in a particular setting.

## Materials and methods

### Study design

All individual studies have received ethical approval from their respective governing bodies. To identify all published reports of PREDSS, a literature search was performed using Medline (PubMed; 1946–2014), OVID, Embase (1947–2014), CINAHL, ISI Web of Knowledge and Google Scholar. Key search terms included: ‘expanded disability status score’, ‘expanded disability status scale’, ‘EDSS’, ‘multiple sclerosis’, ‘self-assessment’, ‘self assessment’, ‘patient reporting’ and ‘self reported’.

These phrases were searched in combination and independently. The outcomes of these searches were inspected by three authors (I.G., C.C. and B.I.) for the inclusion criteria of: (1) patient-reported EDSS, (2) physician-assessed EDSS score and (3) inclusion of all levels of disability. The authors of eligible studies were invited to participate as co-authors, dependent on the availability of their studies’ raw data.

### Statistical analysis

All analyses were performed in SPSS v.22. On receipt of the data, the identity of the studies was masked using a coding system so that the analysis was blinded. The distribution, mean and variance of data from all studies were compared in order to help guide the correct choice of statistical analysis; this was performed visually and using one-way analysis of variance (ANOVA) for means and Levene’s test for variances. Spearman’s rho was used for correlation. Bland–Altman analysis was employed to assess agreement; the gold-standard EDSS was plotted on the *x*-axis. The relationship of EDSS and tool identity with the PREDSS–EDSS difference was explored using analysis of covariance (ANCOVA) within the General Linear Model. For stepwise multivariate linear regression, standard assumptions were met. Significant difference from the null hypothesis was considered to be present when *p* < 0.05.

## Results

### Literature search

The systematic literature search resulted in 423 publications. Eight publications met the inclusion criteria for this study. The first and last authors of each publication were invited to participate by providing a copy of the raw data, which included the physician-assessed EDSS scores, PREDSS scores and functional system (FS) scores. At least one author for each publication responded to the invitation. Data were unavailable for three of the eight studies.^9–11^

### PREDSS tool study characteristics

Table 1 presents the main characteristics of the studies. The tools were developed over a period of 15 years studying a total of 460 patients. Three of the studies deployed their questionnaire directly to the patients in a printed format,^4–6^ while one was assessed using an online electronic format,^8^ and another was deployed via telephone.^7^ The basic design concept is similar among the tools, using a combination of dichotomous, multiple choice or Likert-type questions to assess each of the FS scores within the EDSS, as well as ambulation and dependence on help in daily activities; exceptions are Tool 4 which does not use Likert-type questions, and Tool 5 which includes also some scaling questions asking patients to give percentages. An FS score is generated for each FS, and from this the overall EDSS is calculated. The way in which information about neurological symptoms and functional status was collected differed between tools; this is described in detail in the Supplementary material.

**Table 1.** Characteristics of studies.

	Tool 1	Tool 2	Tool 3	Tool 4	Tool 5	
Reference	Leddy et al.^8^	Bowen et al.^4^	Cheng et al.^5^	Lechner-Scott et al.^7^	Goodin^6^	
Sample size	78	95	147	110	30	
Publication date	2013	2001	2001	2003	1998	
Concordance statistics used	Weighted kappaICC	Percentage agreement	Percentage agreement	KappaICC	None	
		ICC	Kappa			
			Weighted kappa			
			ICC			
Form of tool	Online	Paper	Paper	Phone	Paper	
Number of questions by type:						
*(conditional questions in brackets)*						
Likert	8	16	12	0	23	
Dichotomous	1 (+12)	8	5	2 (+12)	2	
Multiple choice	5 (+9)	10 (+1)	1 (+3)	8 (+5)	6	
Ratio scale	0	0	0	0	(+4)	
Country	UK	USA	USA	Continental Europe	USA	
Multicentre	No	No	No	Yes	No	
Physician EDSS: type	Neurostatus	Kurtzke	Kurtzke	Neurostatus	Kurtzke	
Physician EDSS: standardized training and assessment	Yes	Yes	Probably	Yes	Yes	
Gender (% female)	56%	78%	82%	64%	67%	*p* = 0.08
MS type (% relapsing, versus progressive)	58%	N/A	N/A	42%	53%	*p* = 0.62
Mean age (years)	42	46	42	44	41	*p* = 0.09
Mean EDSS	3.5	4.6	3.4	4.7	4.6	*p* < 0.0005
EDSS variance	1.5	0.7	2.4	0.4	0.4	*p* = 0.002
EDSS range	0–8	0–9.5	0–8.5	0–9	1–8	

ICC: intra-class coefficient; EDSS: Expanded Disability Status Scale; MS: multiple sclerosis.

One-way analysis of variance (ANOVA). Homogeneity of variance tested using Levene’s test.

In all studies, physician EDSS was performed by raters working in the field of MS, in established centres; raters were all trained and assessed, and in two of the studies this was done using a standardized audiovisual package (Neurostatus).^7,8^ Study populations were predominantly female, ranging from 56% to 82%, and approximately half the cases were relapsing–remitting MS. There was no difference between studies with respect to gender, MS type or age. Sample size was similar between studies except for Tool 5 which had a very small sample size of 30 patients. There were significant differences in the mean EDSS and its variance across studies.

## Clinical Scenario 1

### Using PREDSS interchangeably with EDSS: agreement

In Clinical Scenario 1, agreement between EDSS and PREDSS would be needed for interchangeability during data collection, or comparison between datasets. Of the three statistical methods used to assess reliability, the Bland–Altman analysis was considered to be the most suited; it enables direct visualization.

**Figure 1.**
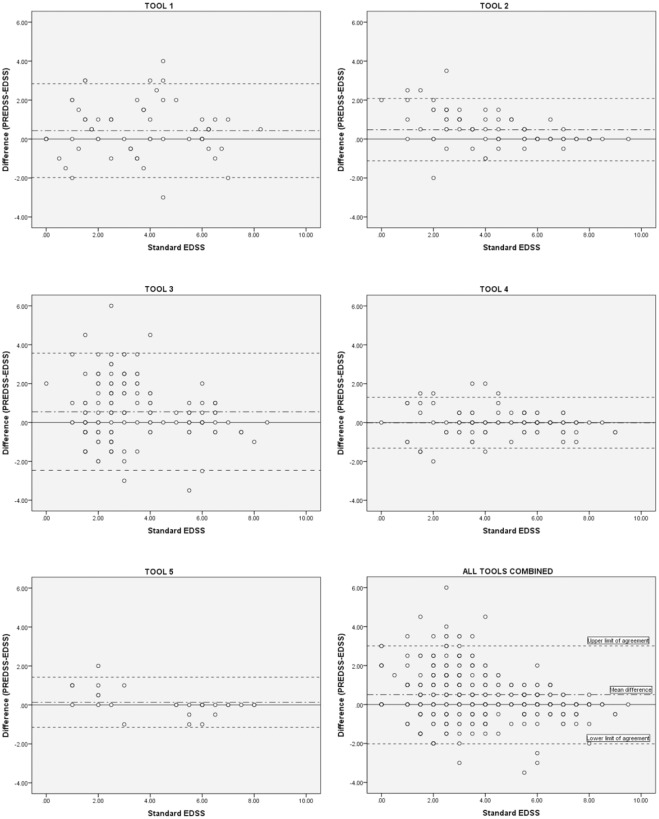
Bland–Altman plots for Tools 1–5, and all tools combined.

Bland–Altman analysis provides a numerical and pictorial estimate of the differences and their 95% limits of agreement. The Bland–Altman plots for EDSS–PREDSS agreement across the whole EDSS range are depicted in Figure 1, which shows a tendency for less agreement at lower levels of disability. The Bland–Altman data, across the whole EDSS range and for EDSS ⩽ 5.5 and > 5.5, are listed in Tables 2–4; this division was necessary since the minimum clinically significant change in EDSS is different in these two disability categories. For EDSS ⩽ 5.5, all the tools overestimated the EDSS (mean difference of all tools combined = 0.51), while for EDSS > 5.5, there was a tendency to slightly underestimate the EDSS (mean difference of all tools combined = –0.02). PREDSS can be corrected for over- or underestimation of the EDSS by subtracting or adding the mean difference respectively, with 95% confidence that the real value of the EDSS lies between the 95% limits of agreement shown on the Bland–Altman plots. Hence, the 95% limits of agreement are more crucial than the mean difference. For all tools, the difference between the 95% limits of agreement exceeded the EDSS change that is considered to be clinically meaningful. Hence, none of the tools can be used interchangeably with the physician-derived EDSS. For EDSS ⩽ 5.5, where a change of ⩾1 is considered to be meaningful, the smallest difference between the 95% limits of agreement was three times higher (3.09, Tool 5). For EDSS > 5.5, where a change of 0.5 is considered to be meaningful, the smallest difference between the 95% limits of agreement was nearly twice as much (0.85, Tool 2).

**Table 2.** Bland–Altman statistics: all EDSS range.

	Tool 1	Tool 2	Tool 3	Tool 4	Tool 5	All tools combined
Reference	Leddy et al.^8^	Bowen et al.^4^	Cheng et al.^5^	Lechner-Scott et al.^7^	Goodin^6^	
*N*	78	95	147	110	30	460
Minimum difference	−3	−2	−3.5	−2	−1	−3.5
Maximum difference	4	3.5	6	2	2	6
Mean difference	0.43	0.48	0.55	−0.01	0.13	0.35
Standard deviation	1.23	0.82	1.54	0.67	0.66	1.15
Upper 95% limit of agreement	2.84	2.08	3.56	1.29	1.42	2.61
Lower 95% limit of agreement	−1.98	−1.13	−2.47	−1.32	−1.15	−1.91
Difference between 95% limits of agreement	4.82	3.21	6.03	2.61	2.57	4.52

EDSS: Expanded Disability Status Scale.

**Table 3.** Bland–Altman statistics: EDSS ⩽ 5.5.

	Tool 1	Tool 2	Tool 3	Tool 4	Tool 5	All tools combined
Reference	Leddy et al.^8^	Bowen et al.^4^	Cheng et al.^5^	Lechner-Scott et al.^7^	Goodin^6^	
*N*	55	63	123	68	17	326
Minimum difference	−2	−2	−4	−2	−1	−4
Maximum difference	4	4	6	2	2	6
Mean difference	0.62	0.71	0.64	0.03	0.32	0.51
Standard deviation	1.30	0.91	1.62	0.81	0.79	1.29
Upper 95% limit of agreement	3.16	2.50	3.83	1.61	1.87	3.03
Lower 95% limit of agreement	−1.93	−1.09	−2.54	−1.55	−1.22	−2.02
Difference between 95% limits of agreement	5.09	3.58	6.37	3.16	3.09	5.05

EDSS: Expanded Disability Status Scale.

**Table 4.** Bland–Altman statistics: EDSS > 5.5.

	Tool 1	Tool 2	Tool 3	Tool 4	Tool 5	All tools combined
Reference	Leddy et al.^8^	Bowen et al.^4^	Cheng et al.^5^	Lechner-Scott et al.^7^	Goodin^6^	
*N*	23	32	24	42	13	134
Minimum difference	−3	−1	−3	−1	−1	−3
Maximum difference	1	1	2	1	0	2
Mean difference	−0.02	0.03	0.06	−0.08	−0.12	−0.02
Standard deviation	0.92	0.22	0.86	0.33	0.30	0.57
Upper 95% limit of agreement	1.79	0.46	1.76	0.56	0.47	1.10
Lower 95% limit of agreement	−1.83	−0.40	−1.63	−0.73	−0.70	−1.14
Difference between 95% limits of agreement	3.62	0.85	3.39	1.29	1.17	2.24

EDSS: Expanded Disability Status Scale.

### Putting PREDSS–EDSS agreement in context: comparison with EDSS inter-rater agreement

To put the PREDSS in context, the agreement between EDSS and PREDSS was compared with published inter-rater and intra-rater agreement data for the EDSS. Out of eight studies,^17–24^ six examined EDSS rater variability across a wide EDSS range.^17,18,20,22–24^ These studies variably reported percentage agreement, ICC and kappa for inter-rater and intra-rater agreement. Table 5 lists the percentage agreement (total, within 0.5, 1 and 1.5 EDSS points), kappa and ICC for all five tools, individually and combined together, as well as the percentage agreement, kappa and ICC between EDSS raters in the published studies identified.

**Table 5.** Percentage agreement and ICC between EDSS and PREDSS (in this study) and between different EDSS raters in published studies.

		Percentage agreement				ICC	Kappa for agreement within 0.5
		Complete	Within 0.5	Within 1	Within 1.5		
PREDSS/EDSS							
Tool 1	Leddy et al.^8^	27	53	74	82	0.84	0.24
Tool 2	Bowen et al.^4^	42	65	82	93	0.89	0.52
Tool 3	Cheng et al.^5^	20	47	61	74	0.69	0.20
Tool 4	Lechner-Scott et al.^7^	49	80	91	97	0.95	0.61
Tool 5	Goodin^6^	57	70	97	97	0.96	0.49
	**All PREDSS**	**35**	**61**	**77**	**86**	**0.85**	**0.39**
Inter-rater EDSS (same seniority of raters)							
	Sharrack et al.^23^	69	89	96	100	0.99	
	Noseworthy et al.^22^	69	N/A	95	N/A	N/A	0.89
	Verdier-Taillefer et al.^24^	34	66	N/A	N/A	N/A	N/A
	Francis et al.^18^	45	65	85	85	N/A	N/A
	Amato et al.^17^	50	75	96	100	N/A	N/A
Intra-rater EDSS							
	Sharrack et al.^23^	63	89	100	100	0.99	
Inter-rater EDSS (mixed seniority of raters)							
	Hobart et al.^20^					0.78	
Intra-rater EDSS (senior rater)							
	Hobart et al.^20^					0.94	
Intra-rater EDSS (junior rater)							
	Hobart et al.^20^					0.61	

ICC: intra-class coefficient; EDSS: Expanded Disability Status Scale; PREDSS: Patient-Reported Expanded Disability Status Scale.

The published percentage inter-rater agreement of the EDSS is superior to the percentage agreement between the EDSS and PREDSS. For instance, agreement occurs within 1 EDSS point between different EDSS raters in 85%–96% of cases, and between EDSS and PREDSS in 77% of cases.

EDSS inter-rater ICC is reported to be 0.94–0.99, but the EDSS/PREDSS ICC varied between 0.69 and 0.96 across the tools, with a combined ICC of 0.85. In most clinical trials, EDSS raters receive formal training. However, in real-life clinical practice the EDSS is more likely to be performed by operators who are variably trained and have different levels of experience. One study has shown that the EDSS inter-rater ICC among raters of mixed seniority falls to 0.78;^20^ this approximates the EDSS/PREDSS ICC. Hence, the inter-reliability between PREDSS and EDSS appears to be similar to the inter-reliability between EDSS-trained clinicians of different seniority.

### PREDSS–EDSS agreement varies with tool identity and EDSS

The Bland–Altman analysis showed different levels of PREDSS–EDSS agreement among studies. In addition, agreement was better at the higher levels of disability. There was not a better agreement at the lower end of the EDSS scale, to indicate a floor or ceiling effect. This suggested that PREDSS–EDSS agreement was dependent on the extent of disability. ANCOVA, using tool identity as a fixed factor and EDSS as a covariate, against the PREDSS–EDSS difference as the dependent variable, showed that both EDSS and tool identity significantly affected the variance in PREDSS–EDSS difference. The contribution of EDSS (4.7%) to the variance of the PREDSS–EDSS difference was *circa* double that of the tool identity (2.6%).

### The contribution of individual FS scores to PREDSS–EDSS agreement

To explore the relative contribution of FS scores to the PREDSS–EDSS difference, the ANCOVA was repeated with tool identity as a fixed factor and EDSS and FS differences as covariates, against the PREDSS–EDSS difference as the dependent variable. Most, but not all cases, had FS score data available (*n* = 383). Tool identity and EDSS maintained a significant relationship with the PREDSS–EDSS difference. The pyramidal, cerebellar and visual FS score differences significantly affected the variance in PREDSS–EDSS difference, indicating that the differences between physicians and patients in the scoring of these domains were contributing to the overall difference in scoring between the PREDSS and EDSS. Stepwise multivariate linear regression of the EDSS and functional score differences against the PREDSS–EDSS difference within individual studies identified the visual domain as the most common FS significantly affecting the PREDSS–EDSS difference, with substantial standardized beta coefficients (Table 6).

**Table 6.** Significant functional system predictors of the PREDSS–EDSS difference after stepwise multivariate regression.

	Tools	Standardized β coefficients
Significant predictors		
Visual FS difference	1, 2, 3	Tool 1: 0.57, Tool 2: 0.26, Tool 3: 0.17
Pyramidal FS difference	3, 4	Tool 3: 0.44, Tool 4: 0.36
Cerebellar FS difference	2	Tool 2: 0.21
Bowel and Bladder FS difference	1	Tool 1: 0.27

PREDSS: Patient-Reported Expanded Disability Status Scale; EDSS: Expanded Disability Status Scale; FS: functional system.

## Clinical Scenario 2

### Using PREDSS on its own: correlation

Clinical Scenario 2, described in the ‘Introduction’ section, does not require agreement between the PREDSS and EDSS. In this scenario, correlation between PREDSS and EDSS would indicate the ability of PREDSS to substitute EDSS, as long as PREDSS is used throughout the data collection, and no external comparison is made to EDSS datasets.

The output of all the PREDSS tools correlated highly with the EDSS (Table 7). The highest correlation coefficients were seen with Tools 2, 4 and 5. Correlation differed markedly across disability categories in most studies. The highest coefficients were seen in Tool 2, which also exhibited least variation of correlation between disability categories.

**Table 7.** Correlation statistics.

	Tool 1	Tool 2	Tool 3	Tool 4	Tool 5	All tools combined
Reference	Leddy et al.^8^	Bowen et al.^4^	Cheng et al.^5^	Lechner-Scott et al.^7^	Goodin^6^	
Correlation coefficient: overall	0.86***	0.938***	0.755***	0.96***	0.962***	0.871***
*Across EDSS severity category*						
Correlation coefficient: EDSS 0–3.5	0.693***	0.524**	0.530***	0.758***	0.557	0.604***
Correlation coefficient: EDSS 4–5	−0.036	0.654**	0.086	0.501*	N/A	0.543***
Correlation coefficient: EDSS ⩾ 5.5	0.350	0.968***	0.595***	0.920***	0.948***	0.831***
*Across functional systems*						
Pyramidal	0.795***	0.671***	0.570***	0.807***	0.825***	0.681***
Cerebellar	0.775***	0.557***	0.086	0.792***	0.629***	0.473***
Brainstem	0.281	0.485***	0.411***	0.645***	0.187	0.382***
Sensory	0.595***	0.652***	0.920***	0.481***	0.623***	0.707***
Bowel and Bladder	0.820***	0.695***	0.714***	0.698***	0.950***	0.695***
Visual	0.249	0.450***	0.375***	0.579***	0.796***	0.351***
Mental	0.672***	0.514***	0.406***	0.590***	−0.044	0.318***

EDSS: Expanded Disability Status Scale.

Spearman’s correlation: *p <0.05; **p <0.005; ***p <0.0005.

In order to determine how FS scores contributed to the correlation between the PREDSS and EDSS, correlation coefficients between patient- and physician-derived scores were computed for all FS scores (Table 7). One-way ANOVA showed that there was a significant difference in correlation coefficients between FS scores (*p* = 0.004). Dunnett’s post-hoc analysis confirmed the mental, visual and brainstem domains as having statistically significantly lower correlation coefficients.

## Discussion

### Clinical Scenario 1

This clinical scenario is where agreement is required between PREDSS and EDSS, that is, when the PREDSS and EDSS are used interchangeably, whether this is a research or clinical service setting.

Bland–Altman analysis showed that most tools performed better at higher EDSS. Using the EDSS score as the gold standard for the measurement of disability throughout its range, three reasons could explain the effect of EDSS on PREDSS scoring. First, PREDSS may be easier to score as disability levels rise, for instance, if patients become more aware of their disability because of having a more severe condition for longer. Second, the use of ambulation capacity in the higher EDSS scoring categories may allow for better performance of PREDSS because patient report of ambulation status better matches physician-assessed ambulation capacity (especially if the latter is derived by asking the patient). Third, EDSS in the range of 0 to 3.5 is particularly prone to inter-rater disagreement compared to the higher range,^19,24^ possibly because the combination of FS scores means there are more opportunities to have a poorer correlation; therefore, the disagreement between PREDSS and EDSS at the low end of the scale may reflect the inherent uncertainty in this region.

Strictly speaking, none of the PREDSS tools can be used interchangeably with the EDSS, since the Bland–Altman 95% limits of agreement were wider than the minimum clinically significant EDSS change; this was the case in all tools, across all EDSS categories. Tool 2, in the setting of an EDSS > 5.5, was closest, giving the user 95% confidence that a corrected PREDSS was within 0.85 EDSS points of the physician-derived EDSS.

Research and clinical service settings are very different. Within most clinical service environments, it is not unusual for individual patients to have their EDSS measured by clinicians with different professional backgrounds and seniority, at different times. The ICC between PREDSS and EDSS approximated or exceeded that reported for clinicians of mixed or junior seniority. Hence, these tools are a realistic choice in clinical settings where regular physician-derived EDSS is not achievable.

It is striking that, at EDSS ⩽ 5.5, across all the tools, PREDSS consistently overestimated the EDSS. PREDSS tools also had a tendency to overestimate FS scores (mean differences ranging between 0.07 and 0.53), except for the visual FS (mean difference of −0.22). It is tempting to speculate on the possibility that PREDSS may be more sensitive than the EDSS, by (1) allowing the patient to report the true extent of disability, outside a face to face setting with their clinician and (2) measuring troublesome symptoms which are not accompanied by abnormalities on neurological examination. This notion may be studied further in future studies by examining the correlation of PREDSS and EDSS with a patient-reported measure such as the Multiple Sclerosis Impact Scale–29 (MSIS-29).^25^

### Clinical Scenario 2

This clinical scenario is where agreement is not required between PREDSS and EDSS, but changes on the two scales need to be comparable, that is, when the PREDSS is used instead of the EDSS and comparability needs to be retained with respect to rate of disability progression (ratio of change), whether this is a research or clinical service setting.

All the PREDSS tools correlated highly with EDSS. It is important to emphasize that correlation is not a measure of agreement;^26^ it tests the presence of a relationship between two variables, and the strength and direction of this relationship. Hence, the high correlation demonstrates that PREDSS can replace the physician-derived EDSS in serial measurements for the sole purpose of ensuring proximity of percentage changes between PREDSS and EDSS, but not absolute values of scores or score differences. Correlation coefficients varied depending on the disability level and therefore one may want to select the tool that best suits their application, using Table 7.

Agreement statistics (used in Clinical Scenario 1) and correlation (used in Clinical Scenario 2) measure different entities.^26^ Hence, if there is high agreement, then correlation must be high, but the reverse is not necessarily true, as happened here. Agreement statistics assess to what extent scoring is identical, while correlation statistics measure the relationship between the scores, irrespective of agreement.

### Future directions

Improved versions of these tools should concentrate on the way that pyramidal, cerebellar, brainstem, mental and visual FS scores are scored, since these domains were identified as significant contributors to disagreement and lack of correlation between the PREDSS and EDSS. Among these, the visual FS deserves most attention, since it performed poorly in both Clinical Scenarios (i.e. agreement and correlation). The identification of the visual FS as a major contributor to disagreement with EDSS presents a real opportunity for improvement of PREDSS tools since a smartphone/tablet-based visual acuity testing app, validated for clinical and community-based practice, is now available.^27^

## Supplementary Material

Supplementary material
