# Supplementary material for: A comparative analysis of Patient-Reported Expanded Disability Status Scale tools
Source: Mult Scler. 2015 Nov 12;22(10):1349–58. doi: 10.1177/1352458515616205 (PMC5015760; doi:10.1177/1352458515616205)
Supplement: Supplementary material [file MSJ616205_supplementary_material.pdf]

Qualitative differences between tools

Table S1 lists the ways in which the tools asked about symptoms in each functional system. In the pyramidal system Tools 2 and 5 asked about weakness in each limb in turn, while in other tools, the question was more generic. In the brainstem domain, diplopia and dysarthria were questioned by all tools, dysphagia was not mentioned by Tool 3, and only Tool 5 asked about vertigo. Facial weakness was included as part of the pyramidal system by Tool 5, within the brainstem domain by Tools 1 and 4, and was not covered by Tools 2 and 3. Most tools asked about sensation as touch, while Tools 2 and 4 also asked about pain (± temperature), effectively covering two sensory modalities. Only Tool 2 assessed visual acuity (by asking the patient to read different font sizes) while the other tools asked about visual problems or impairment. Crucially, only Tools 2 and 5 clarified the need to use glasses if these were usually required. The bowel/bladder and cognitive domain questions were very similar to the corresponding assessment in the EDSS. Tool 1 tended to use specific examples to illustrate symptoms while Tool 3 employed the briefest of questions.

Table S1: Tool interpretation of functional systems

	**Tool 1**	**Tool 2**	**Tool 3**	**Tool 4**	**Tool 5**
	Leddy *et al*[8]	Bowen *et al*[4]	Cheng *et al*[5]	Lechner-Scott *et al*[7]	Goodin[6]
Pyramidal	1 general question	each limb in turn	1 question for arms, 1 question for legs	1 general question	each limb in turn; included facial weakness here
Cerebellar	"tremor or clumsy movements", "problems with balance"; used specific examples	"coordination"	"tremor or shaking", "clumsiness or balance problems"	"tremor or clumsy movements", "problems with balance"; used specific examples	"coordination", "difficulty with balance"
Brainstem	"double vision", "slurred speech", "partial loss of facial sensation", "facial weakness", "swallowing problems"	"speech", "swallow", "eye movements unsteady", "seeing double"; also assessed frequency	"double vision", "slurred speech"	diplopia, dysarthria, partial loss of facial sensation, facial asymmetry, swallowing problems	"difficulty swallowing", "difficulty speaking or with your speech", "double vision", "vertigo or dizziness ie a sense or feeling of motion"
Sensory	"touch"	"pain"," temperature", "touch"; each limb in turn	"touch"	"pain", "touch"	"feeling"; each limb in turn
Bowel & Bladder	very similar to EDSS question	very similar to EDSS question	3 questions to assess problems with bowel, bladder or catherization	very similar to EDSS question	very similar to EDSS question
Visual	asks about "visual problems"; need to correct for refractive error not clarified	assesses visual acuity by reading different sized fonts; need to correct for refractive error clarified	asks about "blurred vision"; need to correct for refractive error not clarified	asks about "visual problems"; need to correct for refractive error not clarified	asks about "visual impairment"; need to correct for refractive error clarified
Mental	very similar to EDSS question; with additional examples	Ask about frequency of problems with "thinking"	1 question	very similar to EDSS question	very similar to EDSS question
